# Analysis of New Orthogonal Transforms for Digital Watermarking

**DOI:** 10.3390/s22072628

**Published:** 2022-03-29

**Authors:** Piotr Bogacki, Andrzej Dziech

**Affiliations:** Institute of Telecommunications, Faculty of Computer Science, Electronics and Telecommunications, AGH University of Science and Technology, Mickiewicza 30, 30-059 Cracow, Poland; adzie@tlen.pl

**Keywords:** watermarking, image protection, steganography, multimedia systems, image processing

## Abstract

The paper focuses on the application of new orthogonal transforms in digital watermarking. Novel types of transforms and their characteristics are presented. Potential methods for watermark embedding and recovery are also proposed. They assume embedding hidden information in the transform domains using the luminance channel of the original image. Image spectra are obtained by dividing the original image into smaller blocks that then are further processed by performing the forward transform operation. A watermark is embedded by modifying the spectral coefficients with relatively low values. Since there are various types of transforms, the latter process is realized in an adaptive manner. The proposed solutions were evaluated by measuring the level of visual distortion with respect to the total size of the inserted data. Additionally, the bit error rate (BER) in the recovery phase is also analyzed. The elaborated methods seem to be useful for applications in digital signal and image processing where high imperceptibility and low BER are of great importance. New orthogonal transforms were proved to be useful in watermarking tasks, and in some cases, they can even outperform the classic DCT approach.

## 1. Introduction

Digital watermarking is a method that consists of inserting hidden information into multimedia content, such as images, videos, or audio recordings. Nowadays, in all areas where these digital materials are transmitted, stored, or processed, it is a crucial task to provide adequate protection and prevent the sensitive or valuable data from unauthorized access, modification, or theft. Watermarking algorithms can address all these problems. In addition, they can be used to detect copyright infringement, undesired manipulations, and distribution. Solutions based on watermarking may be also applied in steganographic communication and for the pseudonymization or anonymization of sensitive and private information.

A watermarking algorithm should be properly designed by taking into account the target application and key features it should provide. The most common characteristic is low visual distortion upon the embedding process as well as high recovery accuracy when the watermark is extracted. It should also guarantee that it will be capable of storing sufficient amount of data to perform all the functions it was designed to realize.

A watermark can be embedded in the spatial or transform domain. Operating in the spatial domain means direct modification of multimedia data, such as, for example, color bands or brightness in case of images. The typical example of this approach is the Least Significant Bit (LSB) [1], where the least significant bits of multimedia objects are replaced with information bits. For instance, image pixel values might be altered by substituting the LSB for the message bitstream values. Transform domain techniques result in the modification of spectral coefficients in the given transform domain. They usually provide better results than the ones that operate in the spatial domain [2,3].

The most common transforms used for watermark insertion are discrete cosine transform (DCT) [4,5,6,7], discrete Fourier transform (DFT) [8,9], discrete wavelet transforms (DWT) [10,11], and Walsh–Hadamard transform [12]. In addition, transform-based methods can be used jointly with other techniques, such as, e.g., discrete fractional random transform (DFRNT) [3] or singular value decomposition (SVD) [13].

Usually, the aforementioned transform types are modified and enhanced in order to improve some aspects of the already known watermarking approaches. On the other hand, sometimes, even completely new transforms are proposed and tested as far as their applicability in various tasks is concerned. As an example, Periodic Haar Piecewise-Linear (PHL) transform was evaluated and confirmed to be useful for watermarking purposes by achieving promising results and even outperforming classic DCT transform in some aspects [14]. Another attempt was preseneted in [15], where multiparameter discrete transforms based on discrete orthogonal polynomials are used for watermark embedding.

In this paper, another set of recently introduced orthogonal transforms has been investigated with regard to their applicability in digital watermarking [16].

The paper is organized as follows. The following section presents the transform types that are analyzed. Section 3 describes the watermark embedding methods used for the assessment of the usability of the transforms being examined. Section 4 contains the experimental results and comparisons of the proposed solutions with the DCT approach. Finally, Section 6 presents the conclusions and future work.

## 2. New Orthogonal Transforms

The transforms analyzed in this paper were first proposed in [16]. The main characteristic of these transforms is that their matrices are orthogonal and symmetric. This makes it possible to use only one matrix for both forward and inverse transform operation. Another key feature is the parameterization in matrix generation process. This allows for the creation of an infinite number of transform matrices and at the same time can result in achieving various spectra depending on the set of selected parameter values. In addition, some parameters may be used as a security feature. For instance, in order to create a sample transform matrix, the user only needs to define its size and first three elements. These three values can be treated as a security mechanism that can prevent unauthorized persons from retrieving or getting access to secret information—for instance, in the form of a watermark embedded in a spectrum of an image. In addition, there are a few methods for matrix generation. The five major ones will be briefly introduced in the below subsections.

### 2.1. Full Non-Exponential Matrix

The matrix A(n) has a square shape and size N×N ($n=\log_{2}N,\;n=1,2,3,\ldots$). This form of matrix has a recurrent structure and can be generated by using the below formula:

(1) $$A\left(n\right)=\left[\begin{array}{cc} A_{1}\left(n-1\right) & \;A_{2}\left(n-1\right)\\ A_{2}\left(n-1\right) & \;-A_{1}\left(n-1\right) \end{array}\right]$$

and

(2) $$A\left(1\right)=\left[\begin{array}{cc} a_{1} & \;a_{2}\\ a_{2} & \;-a_{1} \end{array}\right]$$

A sample matrix for n=2 is presented below:

(3) $$A\left(2\right)=\left[\begin{array}{cc} A_{1}\left(1\right) & \;A_{2}\left(1\right)\\ A_{2}\left(1\right) & \;-A_{1}\left(1\right) \end{array}\right]=\left[\begin{array}{cccc} a_{1} & \;a_{2} & \;a_{3} & \;a_{4}\\ a_{2} & \;-a_{1} & \;a_{4} & \;-a_{3}\\ a_{3} & \;a_{4} & \;-a_{1} & \;-a_{2}\\ a_{4} & \;-a_{3} & \;-a_{2} & \;a_{1} \end{array}\right]$$

For matrices A(n), where n≥2, it is sufficient to select only the first three elements of the first row, and the remaining elements need to satisfy the following relation:

(4) $$a_{i}=\frac{a_{i-2}\cdot a_{i-1}}{a_{i-3}}\;\mathrm{for}\;\;i=4,\ldots ,N\;$$

In this way, the basis sequence consisting of *N* elements (N≥4) is obtained. Then, the following relations hold true:

(5) $$A\left(n\right)=A^{T}\left(n\right)\;,\;A\left(n\right)\cdot A^{T}\left(n\right)=C\cdot I\;,\;\frac{1}{C}\cdot A^{T}\left(n\right)=A^{-1}\left(n\right)$$

where:

$A^{T}\left(n\right)$—transpose matrix    *I*—unit matrix

$A^{-1}$—inverse matrix

*C*—coefficients  $C=\sum_{i=1}^{N}a_{i}^{2}$

Satisfying Condition (4) and following Formula (1) guarantee that the matrix A(n) is symmetric, i.e., the forward matrix is equal to its transpose: $A\left(n\right)=A^{T}\left(n\right)$ and, upon taking into account the coefficient *C*, orthogonal—its rows (and columns) are orthonormal vectors:

(6) $$\langle v_{i},v_{j}\rangle =\left\{\begin{array}{c} 0,\;i\neq j\\ 1,\;i=j \end{array}\right.$$

### 2.2. Full Non-Exponential Matrix with Arbitrary β Parameter

For the non-exponential type of matrix, presented in the previous subsection, for each subsequent group of four elements of basis sequence:

$$a_{1},a_{2},a_{3},a_{4}|a_{5},a_{6},a_{7},a_{8}|a_{9},a_{10},a_{11},a_{12}\left|a_{13},a_{14},a_{15},a_{16}\right|\ldots$$

there is a relation:

(7) $$\frac{a_{i+4}}{a_{i}}=\beta =const\;\mathrm{for}\;\;i=1,2,3,4,\ldots ,N-4\;$$

The coefficient β can be any positive number. Then, each subsequent group of four elements of the basis sequence may be calculated by multiplying the previous group by β satisfying Condition (4).

This way, the basis sequence will have the following form:

(8) $$a_{1},a_{2},a_{3},a_{4},\beta a_{1},\beta a_{2},\beta a_{3},\beta a_{4},\beta^{2}a_{1},\beta^{2}a_{2},\beta^{2}a_{3},\beta^{2}a_{4},\cdots ,\beta^{j}a_{1},\beta^{j}a_{2},\beta^{j}a_{3},\beta^{j}a_{4}$$

where β—positive real number

$j=\frac{N}{4}-1\;,\;N=2^{n}\;,\;n=2,3,4,\ldots$

### 2.3. Full Exponential Matrix

This type of matrix is created when its basis sequence consists of consecutive powers of the real number *a* having the following form:

(9) $$a^{k},a^{k+1},a^{k+2},a^{k+3},\ldots ,a^{k+(N-1)}$$

where:

*a*—any real number, a≠0

*k*—integer, k∈(−∞,+∞)

Therefore, the formula for the generation of the exponential type of matrix A(n) of size NxN, $N=2^{n}$ takes the form of the below recursive relation:

(10) $$A\left(n\right)=\left[\begin{array}{cc} A(n-1) & \;A\left(n-1\right)\cdot a^{2^{\left(n-1\right)}}\\ A\left(n-1\right)\cdot a^{2^{\left(n-1\right)}} & \;-A(n-1) \end{array}\right]$$

where

n=1,2,3,… and $\;A\left(0\right)=a^{k}$, *k*—integer

For n=2, we have:

(11) $$A\left(2\right)=\left[\begin{array}{cc} A(1) & \;A\left(1\right)\cdot a^{2}\\ A\left(1\right)\cdot a^{2} & \;-A(1) \end{array}\right]=\left[\begin{array}{cccc} a & \;a^{2} & \;a^{3} & \;a^{4}\\ a^{2} & \;-a & \;a^{4} & \;-a^{3}\\ a^{3} & \;a^{4} & \;-a & \;-a^{2}\\ a^{4} & \;-a^{3} & \;-a^{2} & \;a \end{array}\right]$$

### 2.4. Sparse Non-Exponential/Exponential Matrix

This type of matrix is created by generating matrices of smaller dimensions and positioning them on the diagonal of the matrix currently being created. The sparse matrix $A_{m}\left(n\right)$ has the following form:

(12) $$A_{m}\left(n\right)=\left[\begin{array}{cccc} A(m) & \;0 & \;0\;\;\cdots & \;0\\ 0 & \;A(m) & \;0\;\;\cdots & \;0\\ 0 & \;0 & \;A(m)\;\cdots & \;0\\ \vdots & \;\vdots & \;\vdots \;\;\ddots & \;\vdots \\ 0 & \;0 & \cdots & \;A(m) \end{array}\right]$$

where

$A_{m}\left(n\right)$—matrix with dimension $N=2^{n}\;,$

n=2,3,4,…

A(m)—matrix (1) or (10) with dimension

$M=2^{m}\;,m=1,2,3,\ldots \;,\;m<n$

For example, for the parameters n=3, m=2 the matrix $A_{2}\left(3\right)$ is

(13) $$A_{2}\left(3\right)=\left[\begin{array}{cc} A(2) & \;\cdots \\ \cdots & \;A(2) \end{array}\right]$$

where

A(2)—is defined either by (3) or (11).

## 3. Watermark embedding

**Figure 1:**
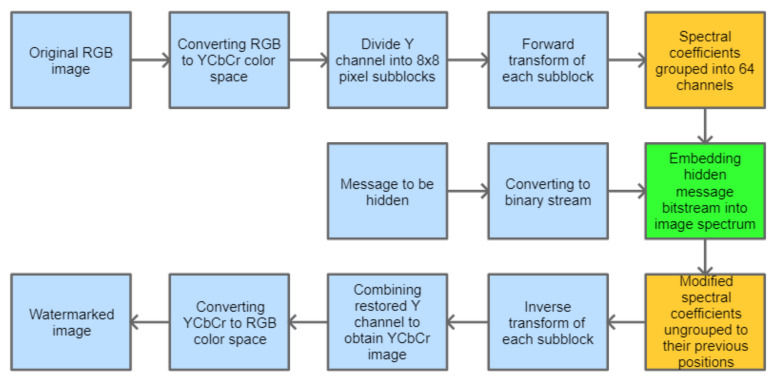
Block diagram for the base process of watermark embedding.

The watermarking algorithm, analyzed in this paper, is based on embedding a random bitstream in the domain of one of the transforms described in Section 2. It is assumed that only the grayscale image, represented by the luminance channel, is processed. As it is usually done, forward and inverse transform operations are performed on smaller subimages, having the size of 8 × 8 pixels. As a result, we obtain a lot of processed blocks, each containing 64 spectral coefficients. This is the standard procedure, which is followed before the subsequent steps are realized. This base procedure for watermark embedding is depicted in Figure 1. The blocks highlighted in orange are optional. The block highlighted in green is the one for which different embedding solutions can be used. Since the aim of this paper is to gain some knowledge about the applicability of the novel orthogonal transforms in digital watermarking, several approaches, both simple and those more sophisticated, are investigated in order to learn which transform types and which embedding methods are the most suitable for specific cases and goals that we want to achieve. Thus, five approaches have been examined and verified. Methods 1 and 4 are classic techniques used as a reference, while methods 2, 3, and 5 are proposed as their adaptive extensions that aim at the efficient use of the presented matrix types. These approaches are presented in the following subsections.

### 3.1. Method 1: Direct LSB Modification of Image Spectrum (M1)

In this approach, the hidden message is evenly distributed in the image spectrum. Channel grouping is not performed; thus, the orange blocks from Figure 1 are skipped. The information bits are inserted into least significant bits of the selected spectral coefficients, similarly as in the JSteg algorithm [17]. This method is very simple, but its main drawback is that it does not analyze the spectrum of an image, and the embedding process for the given message length is always performed in the same way.

### 3.2. Method 2: Adaptive Substitution of Grouped Spectral Coefficients (M2)

This method consists of grouping of the spectrum and then selecting these channels that carry the lowest amount of signal information. Spectrum grouping means that each channel contains all the spectral coefficients that were taken from the same position of each block processed in the forward transform step. This results in 64 transform channels. The further processing is dependent on the nature of the given transform. For instance, the Discrete Cosine Transform has a property of locating the coefficients that represent the low frequencies, to which human eye is much more sensitive, in the top-left channel of the image spectrum with applied grouping [18]. Then, moving across the spectrum in the zigzag order leads to channels representing higher frequencies which are not so noticeable for people. That is why a watermark is inserted in the middle-band frequencies as a trade-off between high robustness and good visual quality. During JPEG compression, the coefficients from the bottom-right channels, representing high frequencies, are removed. However, this paper will not focus on robustness to different attacks such as compression, and for the testing phase, the PNG image format was selected since it supports lossless data compression. Nevertheless, in case of the transforms described in Section 2, their nature may be diverse, and in order to assure that the conditions related to the visual quality and recovery accuracy are met, it is not possible to always follow the same scheme. Therefore, the blocks in which the information bits are inserted are determined in an adaptive manner. This is realized by the calculation of mean values for all the spectrum channels and sorting them in the ascending order. Then, based on this order, the spectral coefficients from the following channels are modified in order to store the information bits. This method consists of replacing the spectral coefficient values with information bit values multiplied by the given constant. Up to some extent, the bigger this constant is, the higher the accuracy in the recovery phase. However, it is accompanied by the higher degradation of the quality of the image with an embedded watermark.

### 3.3. Method 3 (M3): Adaptive LSB Modification of Grouped Spectral Coefficients

In order to improve the quality of the watermarked image and at the same time be able to recover the hidden information with high accuracy, the previous method was slightly modified. In contrast, the information bits are inserted into least significant bits of the spectral coefficients for the given channels. Although it requires casting real spectrum coefficients to integer type and then setting the last bit to zero or one, this approach provides satisfactory results as far as visual perception and recovery accuracy are concerned. Except for the rounding operation, statistically, only half of the least significant bit replacement operations will result in a change of the coefficient value.

### 3.4. Method 4 (M4): Standard QIM Approach

This method also involves the grouping of spectral coefficients. However, the embedding process is different, and its details are presented in [4]. It is based on the quantized projection embedding where randomly permutated columns of a Hadamard matrix are used as base vectors. The projection is quantized with a quantization step based on the default JPEG quantization table. Then, it is used to modify the vector of coefficients from the given block by applying the quantization operator, corresponding to a bit value of the embedded data, for the consecutive frequency bands.

In order to reconstruct the image when all bits are embedded, the transform coefficients need to be reordered back to their previous position, and the inverse transform needs to be applied.

In the extraction phase, all previous steps need to be performed in order to calculate the projection which is then rounded with respect to the given quantization step. When the result is an even number, then the recovered bit is ’0’; otherwise, it is ’1’.

### 3.5. Method 5 (M5): Adaptive QIM Approach

In contrast to the previous method, this approach uses adaptive channel selection for watermark embedding, depending on the transform type, instead of following the zigzag order. This limits the undesired effects observed when applying a zigzag order in the watermark embedding process for the set of transform types described in Section 2. On the other hand, it may not suit so well for DCT transform, since in this case, a zigzag order is preferred.

## 4. Experimental Results

### 4.1. Image Dataset

For test purposes, 32 images from the ’Images 4k’ dataset [19] have been selected. The dataset consists of 2057 files. The selected images represent various visual characteristics in order to generalize the results and make them more reliable. These characteristics include low contrast, high contrast, low brightness, high brightness, and different color distributions (dominant colors and color variety). The width and height of test images were reduced by half, resulting in a 1920 × 1080 size to speed up the calculations and watermark embedding process.

### 4.2. Transforms Selected for the Experiments

The following transforms and their parameters were selected for watermark embedding:

1. Full non-exponential matrix (NONEXP):
  $n=3,\;a_{1}=1,\;a_{2}=2,\;a_{3}=3$
2. Sparse non-exponential matrix (NONEXP_SPARSE):
  $n=3,\;m=2,\;a_{1}=1,\;a_{2}=2,\;a_{3}=3$
3. Full non-exponential matrix with arbitrary β parameter (NONEXP_BETA):
  $n=3,\;a_{1}=1,\;a_{2}=2,\;a_{3}=3,\;\beta =1.5$
4. Full exponential matrix (EXP):
  n=3,a=1
5. Sparse exponential matrix (EXP_SPARSE):
  n=3,m=2,a=1

### 4.3. Transform Preprocessing

In order to provide satisfactory results as far as both high imperceptibility and low error rate in the recovery phase are concerned, each matrix applied for forward and inverse transform operations had to be initially preprocessed. Upon creation of the matrix according to the standard procedure described in Section 4.2, all its elements are divided by the sum of all elements from the first row of the matrix. This results in all matrix elements belonging to the range: <−1,1>. In this way, when calculating the spectrum of an image, all the spectral coefficients will not reach high values, which will prevent the embedded bits from being lost while saving the image upon inverse transform operation. This transform preprocessing is mainly focused on the assurance that the whole message hidden in an image will be fully recoverable.

### 4.4. Algorithm Testing

The main targets for the analyzed watermark algorithms are the high imperceptibility of the watermark embedded in an image, high recovery accuracy, and large capacity of the watermark. Therefore, the Peak Signal-to-Noise Ratio (PSNR) is taken as a measure of perceptual quality, and the bit error rate (BER) indicates the level of incorrectly recovered bits. The measurements of these ratios were performed for watermarks inserted in the domains of transforms listed in Section 4.2.

**Figure 2:**
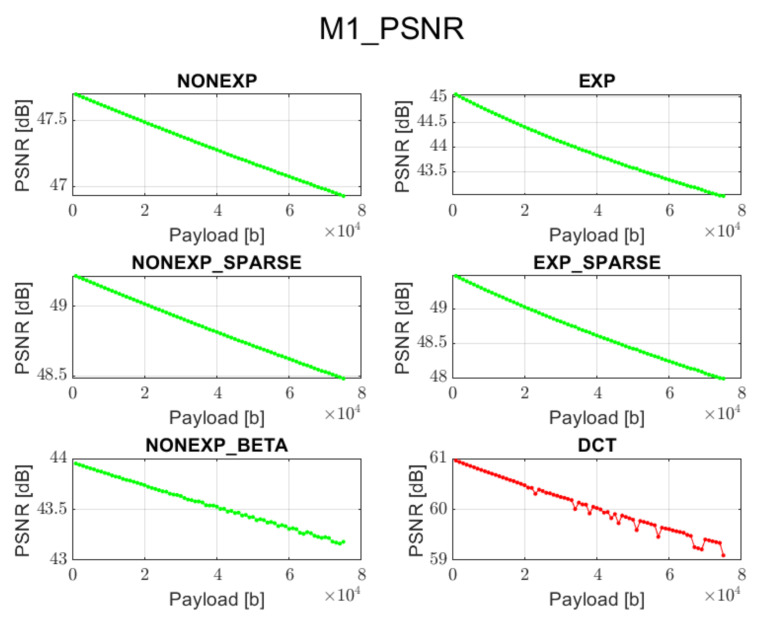
Relation between PSNR and embedded data size for method 1 and all transform types.

**Figure 3:**
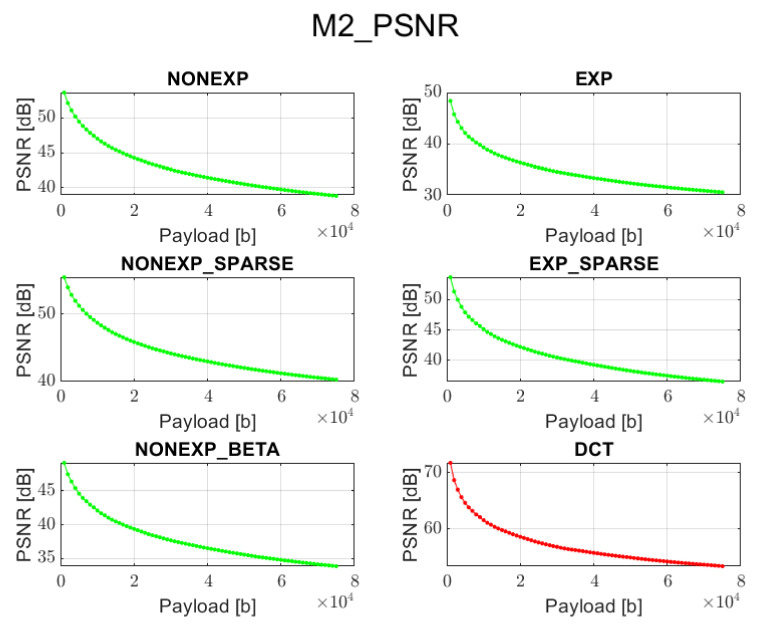
Relation between PSNR and embedded data size for method 2 and all transform types.

**Figure 4:**
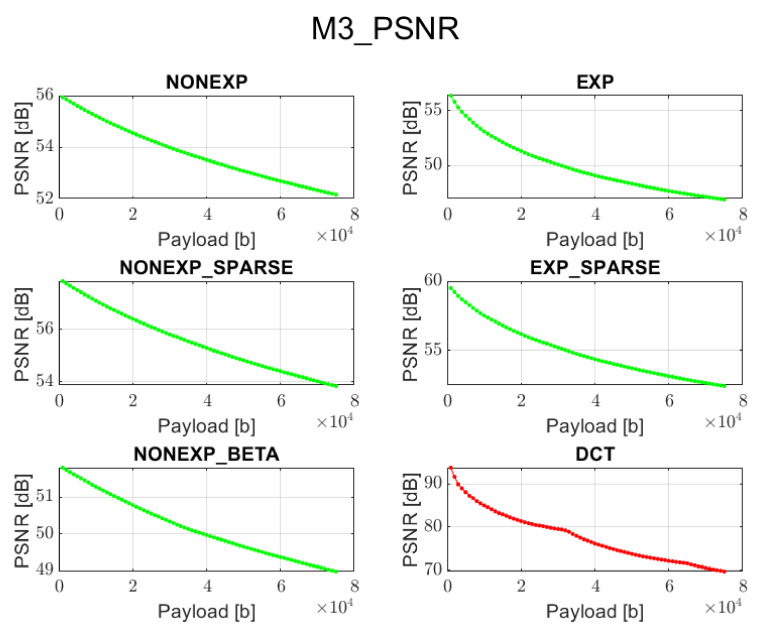
Relation between PSNR and embedded data size for method 3 and all transform types.

**Figure 5:**
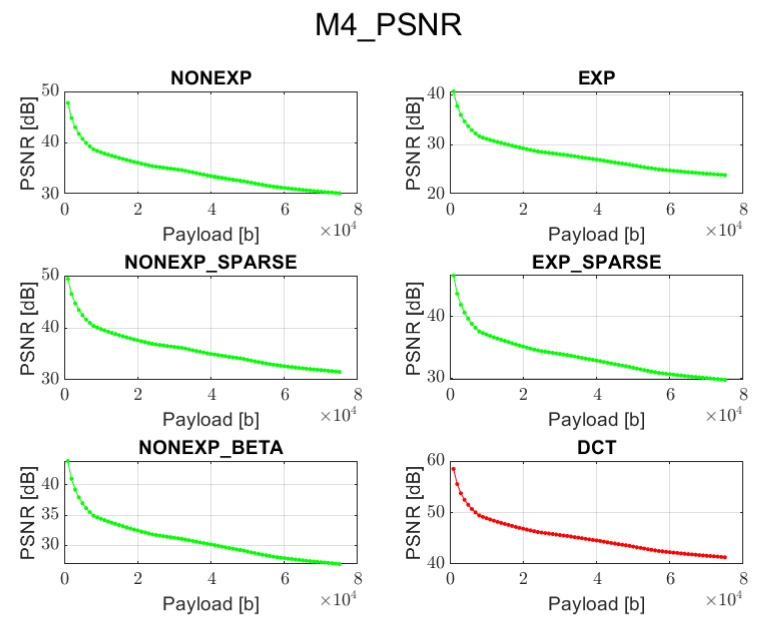
Relation between PSNR and embedded data size for method 4 and all transform types.

**Figure 6:**
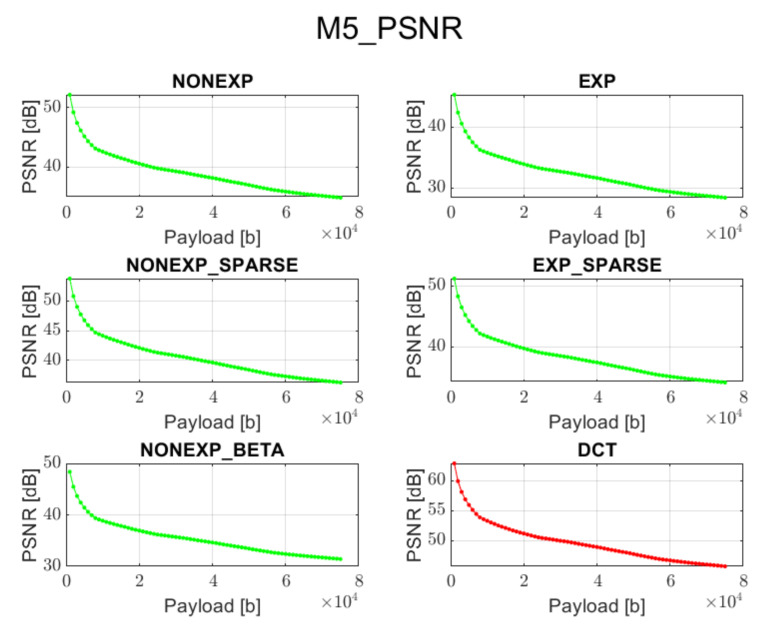
Relation between PSNR and embedded data size for method 5 and all transform types.

The relation between PSNR ratio and the length of the inserted bitstream, for all the above-mentioned embedding methods and transforms, is presented in Figure 2, Figure 3, Figure 4, Figure 5 and Figure 6 (in order to better distinguish both types of transforms, the graphs for the proposed orthogonal transforms are marked in green while for DCT in red). It can be observed that a perceptual quality of an image with a watermark embedded in the DCT spectrum is consistently better than in the case of the remaining orthogonal transforms. However, it is worth highlighting that for methods M1, M2, and M3, the BER is very poor, which means that the watermark cannot be recovered. Thus, the PSNR results in these cases need to be ignored. Only in the case of method M4 is the BER for DCT very low, so only the corresponding PSNR value may be used as a reference. Nonetheless, both transforms can provide satisfying results. It is assumed that the PSNR above 35 dB for 8-bit images indicates that the two images being compared are visually identical, with no perceptual loss of quality [20]. In most cases, such results are achieved, and a PSNR below 35 dB is observed only in case of longer bitstreams or embedding approaches, which apparently do not fit the given transform.

**Figure 7:**
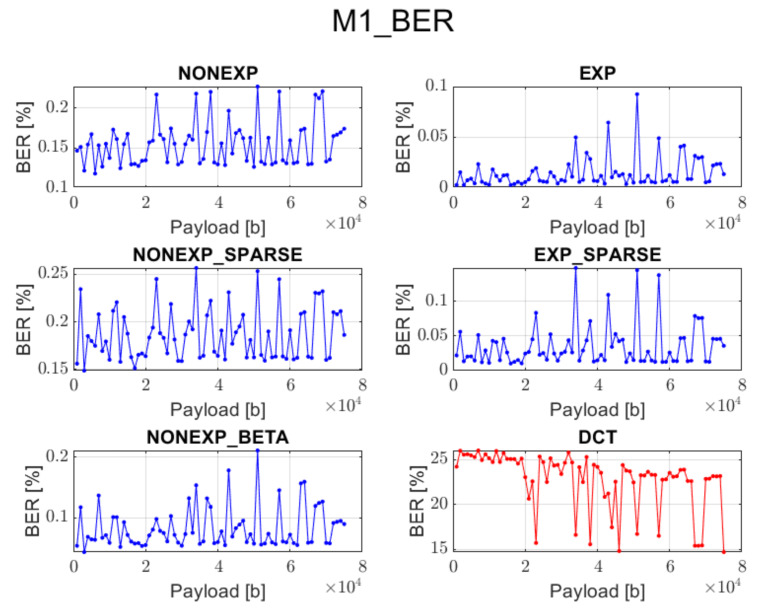
Relation between BER and embedded data size for method 1 and all transform types.

**Figure 8:**
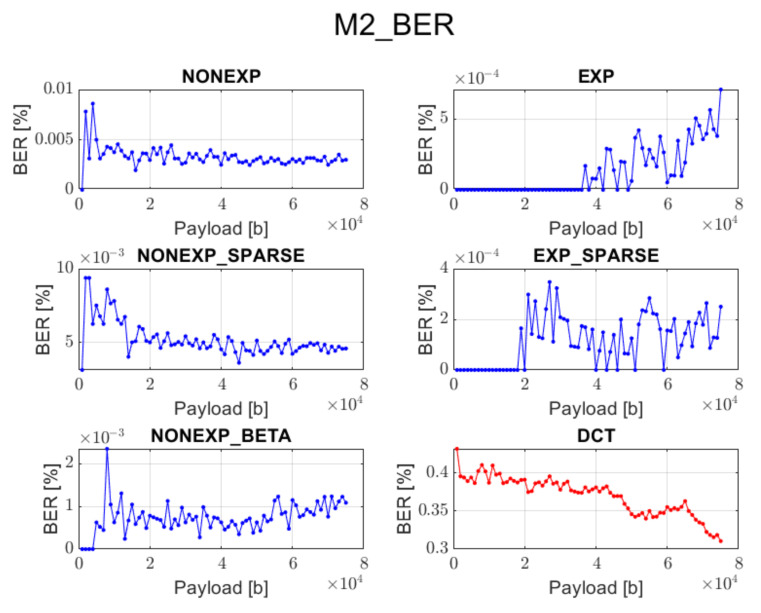
Relation between BER and embedded data size for method 2 and all transform types.

**Figure 9:**
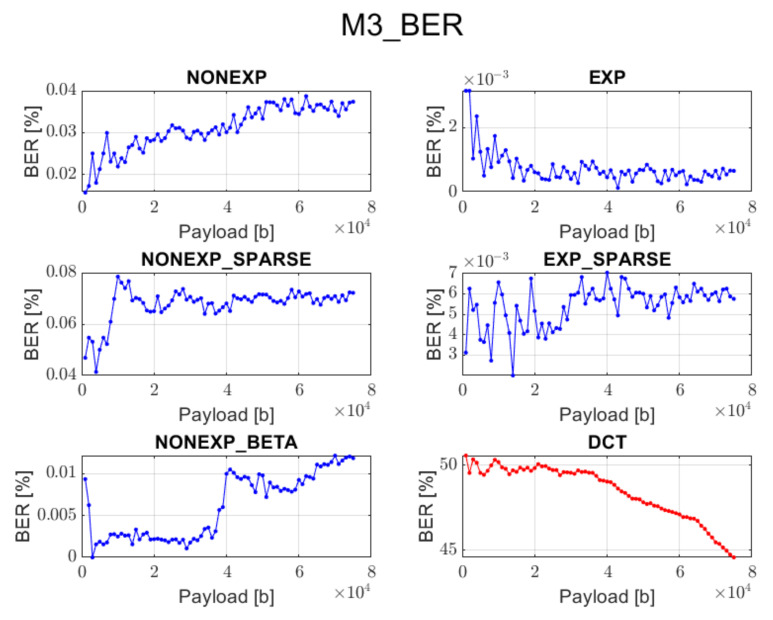
Relation between BER and embedded data size for method 3 and all transform types.

**Figure 10:**
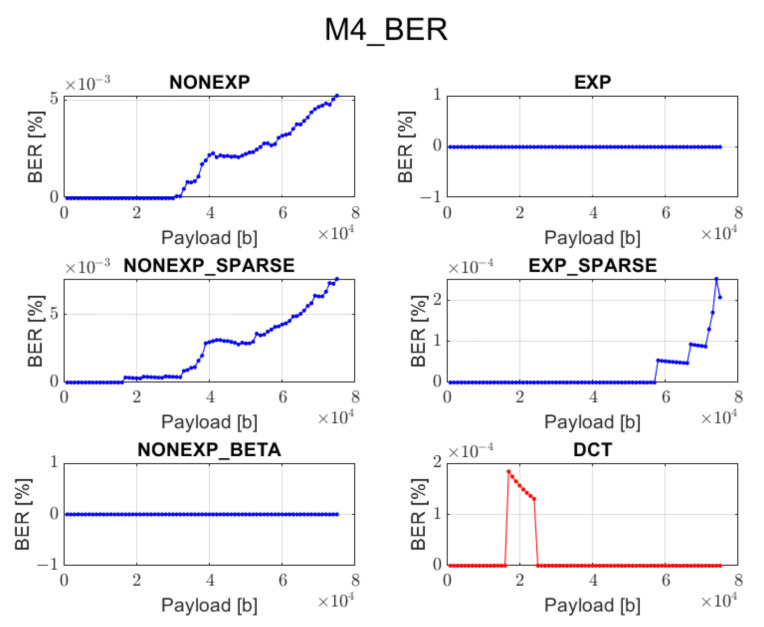
Relation between BER and embedded data size for method 4 and all transform types.

**Figure 11:**
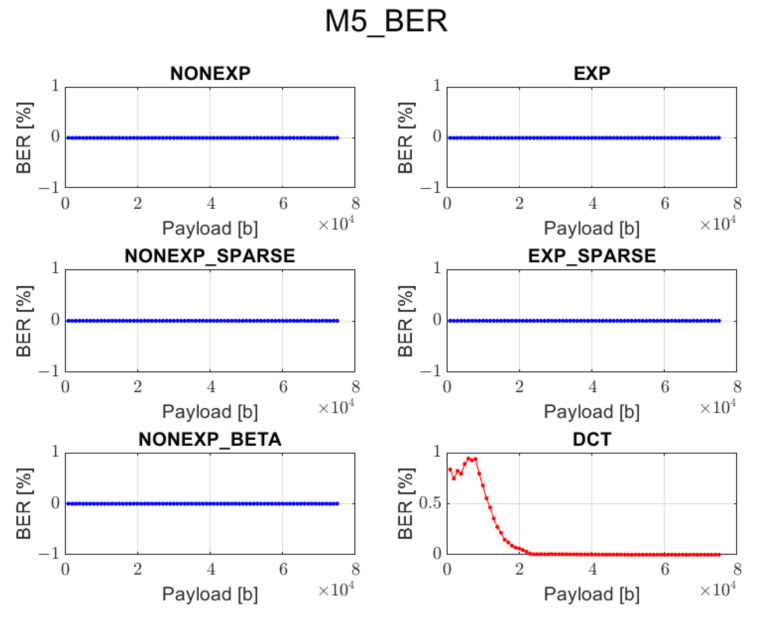
Relation between BER and embedded data size for method 5 and all transform types.

The relation between BER ratio and the length of a hidden message, for particular embedding approaches and transforms, is shown in Figure 7, Figure 8, Figure 9, Figure 10 and Figure 11 (in order to better distinguish both types of transforms, the graphs for the proposed orthogonal transforms are marked in blue while for DCT in red). As can be seen, some methods do not guarantee a sufficient level of accuracy during the extraction process. It is understandable in the case of Method 1 since it uses even distribution during the embedding stage. For proposed orthogonal transforms, methods 2, 3, and 5 provide adaptive embedding, which selects these blocks of spectral coefficients that should not degrade the original image and at the same time allow for almost full data recovery. The BER does not exceed 0.1% in most situations, with the total length of the watermark bitstream ranging from 1000 to 75,000 bits. When using method 4 or 5, the BER is predominantly equal to zero. The DCT approach provides the most reliable and acceptable results for method 4. This could be predicted due to the fact that this method uses a zigzag order for channel selection during the watermark insertion phase, which is specifically designed for this type of transform and is also used in JPEG compression [21]. Although a watermark inserted in the presented orthogonal transform domains seems to fit better in different embedding approaches, both transform types are useful when a large amount of information needs to be inserted into an image.

**Figure 12:**
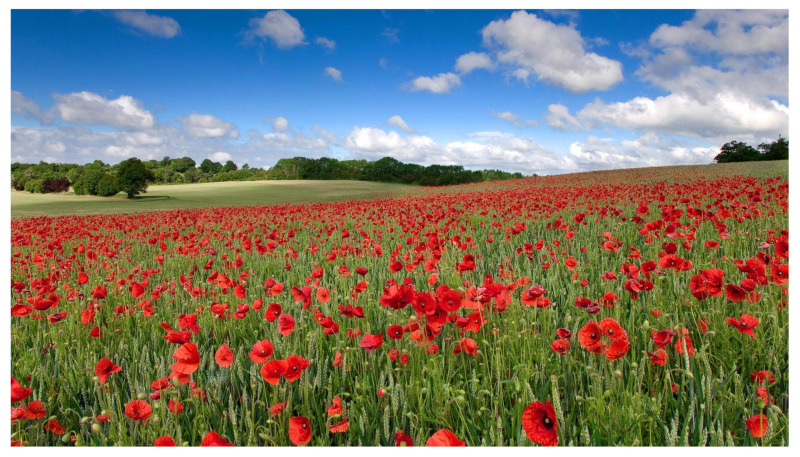
Sample watermarked image obtained by using method 3 and sparse exponential matrix for the message length equal to 50,000 bits.

As an example, the watermarked image for one of the test images is shown in Figure 12.

## 5. Summary of Results

Based on the performed experiments, we can conclude the following:

- Both approaches provide satisfying results as far as both high imperceptibility and data recovery accuracy are concerned.
- All presented types of transforms may be used to embed a watermark of high capacity.
- The DCT method works well only when using method 4 for data embedding, and then, it provides better visual quality and comparable accuracy of information recovery in comparison with the proposed transforms.
- The proposed orthogonal transforms provide satisfactory results for both PSNR and BER when using methods 2, 4, or 5 and for some cases also method 3.
- The DCT method cannot guarantee data recovery during the extraction process in case of methods 1, 2, 3, and 5.
- Spectrum analysis and adaptive watermark embedding guarantees that the proposed orthogonal transforms can provide promising results and may be found useful in many potential applications.
- Matrix parameterization is an interesting feature that might be used as a security mechanism for both data embedding and recovery.

Since the proposed and DCT transforms have their unique nature, in order to compare these two approaches, the concrete combinations of watermark embedding methods and types of transforms have been selected.

1. For proposed orthogonal transforms: method 3 and sparse exponential matrix of size 8 × 8 pixels.
2. For DCT transform: method 4 and DCT matrix of size 8 × 8 pixels.

**Figure 13:**
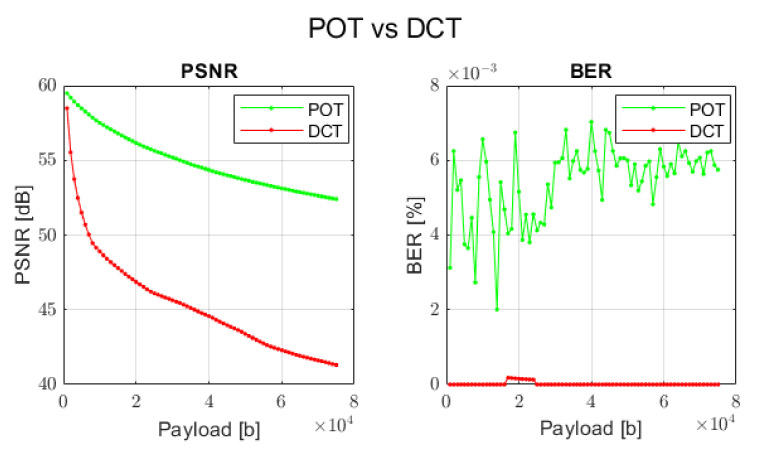
Comparison between proposed orthogonal transform (POT) and DCT transform with respect to PSNR and BER.

The comparison results are depicted in Figure 13.

As can be seen, the proposed orthogonal transforms can even outperform the DCT approach when the visual perception is our main goal. Conversely, if the main target of the application is to guarantee full data recovery and some level of image distortion is acceptable (but still satisfying), one can apply a different type of the proposed transforms with another embedding scheme. This is especially noticeable in case of method 5 and all types of transforms. This means that depending on the task one wants to realize, an adequate procedure can be selected in order to provide expected results.

## 6. Conclusions and Future Work

We have presented novel watermarking algorithms that utilize new types of orthogonal transforms. The elaborated methods satisfy the initially defined requirements as far as high perceptual quality and low BER are concerned. Simultaneously, they provide a high capacity of the inserted information. Moreover, the new orthogonal transforms in most cases provide better results than those of the DCT approach. All these features result in a wide range of potential applications in multimedia systems and services.

Further steps will include analysis of the algorithm behavior in the presence of various attacks and finding potential improvements in order to assure a sufficient robustness level. Moreover, it is planned to carry out research on the spectrum shaping by the selection and optimization of the transform parameters.

## Data Availability

Not applicable.
